# First-Time Parents’ Support Needs and Perceived Support From a Child Health Service With the Integrated New Families Home Visiting Programme

**DOI:** 10.1177/10497323231208972

**Published:** 2023-11-03

**Authors:** Kristin Marie Sæther, Bettina Holmberg Fagerlund, Kari Glavin, Nina Jøranson

**Affiliations:** 1 Faculty of Health Sciences, 87368 VID Specialized University, Oslo, Norway

**Keywords:** first-time parents, child health service, New Families home visiting programme, parental self-efficacy, transition to parenthood

## Abstract

The transition to parenthood is complex and influenced by interacting factors related to society, the parents and the child. Professional support is considered to be one of the societal factors affecting this transition by facilitating parents’ sense of confidence and their competence as parents. In this study, we aimed to explore first-time parents’ support needs and experiences of support from a child health service with the integrated New Families home visiting programme, in the context of their transition to parenthood in the first year postnatally. Interpretive description guided this qualitative study. Six couples and one mother, all well-educated and employed, were interviewed individually (*N* = 13). The parents were recruited from the research project ‘New Families – Innovation and Development of the Child Health Service in Oslo’. We found that being a first-time parent is perceived as overwhelming and that defining support needs may be challenging, particularly in the first period postnatally. In addition, the support needs are constantly changing due to the complexity of interacting factors and the ongoing development of confidence in the parental role. Retrospectively, the parents were satisfied overall with the support from the child health service. The home visit during pregnancy facilitated management and relational and informational continuity. However, we identified a need for even more proactive information provision and communication to optimise the service’s availability and efficiency postnatally. In addition, the importance of peers and of approaching both mothers and fathers must be acknowledged in facilitating parental confidence.

## Introduction

The transition to parenthood is influenced by interacting factors related to society, the parents and the child (Fang et al., 2021; Taraban & Shaw, 2018). Further, parental confidence is found to play a bidirectional role in this complex interplay of factors (Bandura, 1997; Sæther et al., 2023). Professional support from services in the municipalities and hospitals is considered one of the societal factors that aims to smooth the transition to parenthood by facilitating parents’ sense of confidence and their competence as parents (Finlayson et al., 2020; Leahy-Warren et al., 2005; McLeish et al., 2020; World Health Organization, 2022).

Sense of confidence in the parental role as a concept is used interchangeably with parental self-efficacy (PSE) in the literature (Sæther et al., 2023; Vance & Brandon, 2017). In this study, we equate the use of parental confidence and PSE and define PSE as parents’ belief or confidence in their ability to carry out parenting tasks successfully and as a distinct, domain-specific concept captured under Bandura’s self-efficacy (SE) theory (Bandura, 1977, 1997; Vance & Brandon, 2017). This theory outlines four sources which inform SE. ‘Enactive mastery experiences’ serve as indicators of capability; ‘vicarious experiences’ can have an impact through the transmission of competencies and comparison with others; ‘verbal persuasion’ refers to the social confirmation that a person possesses certain capabilities; and a person’s ‘physiological and affective states’ can affect how they judge their own capabilities (Bandura, 1997).

PSE is considered a strong predictor of parental functioning, affecting both the parent’s and the child’s well-being (Albanese et al., 2019). In the first period after birth, first-time mothers are found to have lower levels of PSE than multiparous mothers, and first-time fathers are found to be less confident than first-time mothers (Salonen et al., 2009). Bandura (1997) has argued that, as humans, we can have different levels of SE in different situations or tasks within a domain. Differences in PSE between two parents may affect their cooperation in parenting, and good cooperation is found to facilitate PSE (Favez et al., 2016; Feinberg, 2002).

In family-related interventions and research, PSE is a common outcome measure (Liyana Amin et al., 2018; Wittkowski et al., 2017). Realistic information, emotional and practical support, continuity in and between services, and access when needed are all characteristics of professional support identified as facilitating PSE. PSE is also facilitated by a trusting relationship, the inclusion of both parents, individualised care and friendly professionals collaborating to identify parents’ strengths and needs (Sæther et al., 2023). These characteristics align with the concepts of management, and informational and relational continuity, which are key functions and effective in accommodating parents’ support needs (Barimani & Vikström, 2015; Haggerty et al., 2003, 2013).

The home visit as a form of professional support is recommended in maternal care by the World Health Organization (2022). Home visits are found to facilitate a trusting relationship between the parents and the public health nurse (PHN; Aston et al., 2015), to promote parental confidence (Bäckström et al., 2021; Glavin et al., 2023) and to enhance the involvement of fathers (Solberg et al., 2022). Yonemoto et al. (2021) found in their review that increasing the number of home visits may promote infant health and exclusive breastfeeding. Further, home visits may improve maternal satisfaction with care when compared to hospital check-ups. However, they conclude that the findings are inconsistent and that more research is needed.

The Norwegian healthcare service is based upon a social-democratic welfare state model (Esping-Andersen, 1990), also called the Nordic model. This model is characterised by free public services funded by a tax system to promote fair distribution among social groups and a high level of trust in the services among the general population. In Norway, a national child health service (CHS) offers a universal, standardised programme with age-specific consultations at child health clinics (CHCs) and a home visit 7–10 days after discharge from the hospital (see Table 1). Approximately 99% of children between 0 and 5 years of age and their families use the CHS (Statistics Norway, 2023), which is regulated by law and free of charge. The CHS programme aims to facilitate mastery in the parental role; promote good interaction between parents and children; support children’s development; prevent and detect violence, abuse and neglect; identify developmental anomalies; and help children in receiving necessary follow-up and referrals when required (Norwegian Directorate of Health, 2017).

**Table 1.** Description of the Regular National Child Health Service Programme and the New Families Home Visiting Programme.

*Follow-up by general practitioner and/or midwife during pregnancy*	
Regular national CHS programme	New Families home visiting programme
• For 0- to 5-year-old children and their families	• For parents from the 28^th^ week of pregnancy until their child is 2 years old
• One home visit 7–10 days after discharge from hospital, followed by 13 consultations at the local CHC	• Number of home visits and agenda adapted to parents’ needs
• Agenda adapted to parents’ needs but also predetermined themes and procedures, such as vaccination	• Direct telephone number for PHN
• Drop-in service once or twice a week, initiation of postnatal maternity groups	• Extra consultations at the CHC instead of home visits, if parents prefer
• PHN conducting the regular national CHS programme	• PHN conducting the NF programme based on the programme manual, mandatory training and guidance from a trained PHN
*Both programmes provided by the same PHN (primary nurse model)*	

*CHS, child health service; NF, New Families home visiting programme; PHN, public health nurse; CHC, child health clinic.

A supplementary programme, ‘New Families’ (NF), was integrated into the CHS in all city districts of Oslo between 2016 and 2019 (see Table 1). The programme is based on home visits by a PHN from week 28 of pregnancy until the child is 2 years old. The purpose of NF is to establish a relationship between the family and the PHN during pregnancy, to strengthen the new parents’ confidence and ability to cope with their parental role, and to promote the family’s health. In the long term, the programme aims to bolster children’s readiness for school (City of Oslo, 2018). The programme was developed in a district with a high percentage of immigrants and the highest poverty rate in Norway (Leirbakk et al., 2018, 2019, 2023) before being integrated as a universal offering for first-time parents, parents expecting their first child together and parents having their first child in Norway. NF has a salutogenic perspective, focusing on resource mobilisation, SE and communication methods to facilitate a positive, dynamic dialogue. The number of visits is not standardised because the offering is intended to be tailored to the family’s needs. The same PHN provides both programmes in the form of a primary nurse model (City of Oslo, 2018; Leirbakk et al., 2018, 2019, 2023).

The current study is a qualitative sub-study within the larger, ongoing research project ‘New Families – Innovation and Development of the Child Health Service in Oslo’ (National Library of Medicine, 2019), investigating the effects of, experiences with and implications of NF. The current study aimed to explore first-time parents’ support needs and perceived support from a CHS with the integrated NF home visiting programme, in the context of their transition to parenthood in the first year postnatally.

## Method

### Methodology

Interpretive description (ID; Thorne, 2016) guided this study. This methodological approach is inductive and seeks to generate an understanding of clinical phenomena relevant to the practice through a disciplinary lens (Thorne, 2016). By replacing the rules and structures of traditional methodologies with relevant and meaningful disciplinary logic, we can, according to ID, generate new insights and translate them into practice. However, this requires an actual real-world question, an understanding of existing empirical evidence and a conceptual and contextual understanding of the field. ID has no uniform strategy for data analysis but highlights a reasoning process to work out a design logic to answer the research question (Thorne, 2016). We chose individual, semi-structured interviews as a method to explore the parents’ experiences and Graneheim and Lundman’s (2004) content analysis as a framework to guide the first steps in our analysis.

### Setting and Recruitment

The participants in the current study were recruited from the intervention group in the larger research project. The intervention group consisted of first-time mothers and their partners from three different city districts participating in the regular CHS programme and the NF home visiting programme. Despite efforts to recruit a diversity of participants to the larger research project, most participants were partnered, had a high educational level and were ethnic Norwegians (Brekke et al., 2023). Inclusion criteria in the present study were couples in the intervention group who had answered the final questionnaire in the research project 12 months postnatally – between March and June 2020 – and who had consented to be contacted for further research. An information letter detailing the study’s objective and information about withdrawal was sent by mail, together with a new consent form. Twelve couples met the inclusion criteria, and six couples and one mother agreed to be interviewed. Reasons for not participating were not responding and not prioritising due to time constraints and interviews being too personal in nature.

### Description of the Participants

The participants were between 29 and 48 years of age (mean of 35.4 years), had higher education, were either cohabiting or married, were employed and had a Norwegian or Nordic country background. Their children were between 17 and 21 months old when the interviews were conducted. The distribution of parental leave within couples and in combination with work differed in each family, and most families did not have grandparents living nearby. Five of the seven families had received a home visit from the NF programme during pregnancy, and three of the six fathers had participated in that visit. All the families had followed the regular CHS programme through the first year and received a home visit approximately 1 week after discharge but no further home visits from the NF programme postnatally.

### Data Collection

A semi-structured interview guide was used to collect data. We asked three open questions: How did you experience becoming a mother/father? How would you describe yourselves as parents? What are your experiences with the CHS, particularly the NF programme and home visits? The interview guide contained follow-up questions that allowed the interviewer to explore the material in greater depth. All the parents were interviewed separately in 13 individual interviews. The first author conducted the interviews in November and December 2020 by telephone due to COVID-19 restrictions. The interviews lasted between 27 and 56 minutes (mean of 42 minutes) and were audio recorded with a device not connected to the internet. The interviews were transcribed verbatim.

### Analysis

The analysis process commenced with data collection, and one interview informed the next. Inspired by Graneheim and Lundman’s (2004) content analysis approach, we read through the transcriptions several times, searching for manifest and latent content for interpretation. Both manifest and latent content require interpretation but may vary in depth and level of abstraction (Graneheim et al., 2017). For instance, the research question regarding the service provided data on a more specific level than some of the questions related to parenting. This required different levels of interpretation within our data set. We identified meaning units, condensed the units, identified codes and synthesised the codes into subcategories and one overarching category (see Table 2). This analysis process produced discussions and several revisions before the analysis was finally completed.

**Table 2.** Examples From the Different Steps of the Analysis.

Category	A dynamic development of parental confidence		
Subcategory	Information needs	Co-parenting	Mastery process
Code	Expectations versus reality	We-relationship	Sharing ups and downs
Condensed meaning unit	People don’t give realistic information about the first period of parenthood. Everything is new – e.g., you don’t know how to soothe the baby when it is crying	Great being together in caring for the little baby	Wry look and humour around good and bad mastery experiences in the maternity group
Meaning unit	It’s likely that people don’t always tell you how the first period might be. Everything is new, and you don’t know up from down where the baby’s concerned or why it is crying. Is it hungry? Does it need a new diaper? And when you have tried everything, and the baby is still crying, then you’re like, what do I do next, what next? (Mother)	I actually think it was incredibly sweet and lovely that we, the two of us, were together by ourselves with this little baby. (Mother)	[In the maternity group] there was probably a bit of a wry look at motherhood and a bit of ‘gallows humour’ around what you actually mastered and mostly everything you didn’t master. (Mother)

When performing content analyses, it is common to organise the interpretation of the manifest content into subcategories and categories and the latent content into subthemes and themes (Graneheim et al., 2017; Graneheim & Lundman, 2004; Lindgren et al., 2020). However, we chose to integrate all the content within three subcategories to shape a holistic understanding of the findings. Further, we identified a common thread running through the data, which became the overarching category. These last steps of the analysis required a reorganisation and re-contextualisation of the findings, leading to a higher level of abstraction than being close to the text (Graneheim et al., 2017; Lindgren et al., 2020).

### Ethical Considerations

The study was designed and conducted in accordance with the Declaration of Helsinki (World Medical Association, 2013). Both oral and written informed consent to participate in the interview study was collected from all participants, and data were stored via the Service for Sensitive Data (University of Oslo, 2016). The overall project was approved by the Regional Committees for Medical and Health Research Ethics (reference number: 2018/1378) and the Norwegian Agency for Shared Services in Education and Research (project number: 735207), and it was registered at ClinicalTrials.gov (NCT04162626).

## Findings

### A Dynamic Development of Parental Confidence

**Figure 1.**
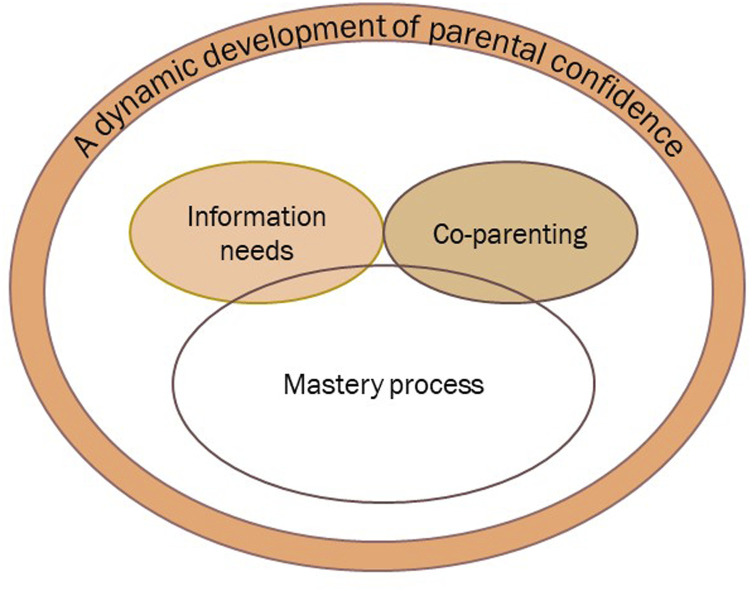
Overarching category and subcategories.

The overarching category ‘A dynamic development of parental confidence’ captures the parents’ development from being insecure in their parental role to having a strong belief in themselves as parents. This development was not a conscious process, and several found it interesting to reflect on this process during the interviews. The development of confidence is described through the parents’ experiences of support needs and perceived support in the first year postnatally, which are presented in the subcategories. The first subcategory, ‘Information needs’, represents the first period in which everything is new and overwhelming. In this period, support needs might be indistinctive, affecting the parents’ use of professional support. ‘Co-parenting’ represents the importance of mutual support from one’s spouse, parents experiencing themselves as one unit and fathers’ desire for involvement in childrearing and for inclusion in the CHS. The last subcategory, ‘Mastery process’, represents the constant and ongoing learning process around being a parent, informed by previous and ongoing mastery experiences and strategies, responses from the child and support from peers and professionals. ‘Information needs’ and ‘Co-parenting’ are independent subcategories; however, they do also inform the subcategory ‘Mastery process’ as a kind of context or background (see Figure 1).

#### Information Needs

Despite extensive preparations ahead of birth, including learning a great deal about baby care, once the baby was born, “everything had to be done for the first time by the mother, the father and the child” (Father). While a couple of the mothers had previously looked after children in the context of work or family, none of the fathers had this experience. Many fathers said they relied on and learned baby care from their partners. The emotional changes were the biggest surprise for the parents, in the gap between expectations and reality in the first period after birth.

It’s a completely crazy adjustment – very nice, very exhausting. You feel that you suddenly have more emotions than you’ve had before ... A mixture of several emotions – actually, all the emotions ... No, I don’t think I’ve ever been so exhausted before, but at the same time so happy. (Mother)

Retrospectively, the parents described the first year as manageable overall, and they saw the challenges as “something most people struggle with” (Father). However, in the midst of a challenge they often felt insecure. Some parents had received realistic information about being a parent, while others felt that the disadvantageous aspects of becoming a parent were not touched upon and that this challenged their feelings of normality. Despite being prepared that lack of sleep would be an issue, some fathers were surprised that their sleep would be so fragmented and affect their performance at work. Several mothers described tiredness as less prominent in the first period. However, lack of sleep over time (i.e. months) was experienced as more challenging. A child’s illness created insecurity in both parents, and strong feelings arose when their baby became sick, as well as when it recovered. Further, general restlessness and the need for long-lasting and intense physical contact were mentioned, as well as breastfeeding and food challenges. Two of the mothers ended breastfeeding earlier than they intended. One expressed great disappointment at her inability to breastfeed. She had been unaware of the additional breastfeeding support she could have received at her local CHC. In addition, she had feared being negatively judged by the PHN. In retrospect, this fear was seen as unfounded because the PHN had been supportive. Another mother highlighted the need for clearer information about when and how to contact the CHC, especially when distraught and in need of psychological support.

The prenatal home visit from the NF programme was generally found to be a highly positive experience.

The home visit before birth was really nice because someone we knew came to our home right after the birth. Yes, getting to know each other and having a familiar face when I started knocking on their doors (visiting the CHC often) was very good. (Mother)

Despite it being a positive experience, several parents were uncertain about the purpose of the prenatal visit, not knowing what they should or could ask for. In addition, two families did not receive the prenatal visit. One family had to cancel because the mother was in hospital, having delivered the baby. The other family was not informed about the opportunity to have a prenatal home visit, neither by the mother’s general practitioner (GP) nor the midwife. However, the mother thought a prenatal home visit would have been a beneficial opportunity as a final check-up before entering motherhood.

Some fathers were not aware that postnatal home visits had been a common offering in Norway for many years. The home visit during the first week postnatally was appreciated by both parents. However, uncertainties related to needs and possible questions were again addressed. Uncertainties were even reinforced by fluctuating emotions and perceptions like “my mind was somewhat foggy” (Mother). Some described home visits in general as less clear and more unplanned than the consultations at the CHC, and one father called for a clearer agenda and more management from the PHN.

All the mothers and fathers were in favour of having the opportunity to receive many home visits if needed. However, the threshold for actively asking for a home visit was perceived as high, due to uncertainty around whether their challenges qualified for home visits versus drop-in visits or consultations at the CHC. Home visits were also perceived as the PHN’s opportunity to check the home, and the parents reflected on the PHNs’ dual mandate in giving parental support and controlling the child’s environment. This control function was perceived as an important societal mission because “there are a lot of weird things out there” (Father). None of the parents expressed concerns about being negatively judged.

#### Co-Parenting

Both mothers and fathers often used ‘we’ instead of ‘I’ during the interviews, referring to themselves and their partners. They were proud of what they had achieved together, acknowledged each other’s cooperation and support and described having confidence in each other’s parenting skills. Many fathers acknowledged the mother as most important for the child, particularly during the initial period. Nevertheless, all the fathers wanted to have a central position in raising their children, which the mothers also expected and desired.

It’s crucial to give space to the father ... he is equally important, even though the mother possesses breasts. The father can change nappies, play with and rock the baby, carry the baby in a carrier or a sling and take turns getting up at night. (Mother)

Some fathers highlighted that the role of the father had changed from previous generations, and they saw themselves as pioneers. A couple of fathers switched to jobs that involved less travelling during this first year. The travelling was described by one father as a double-edged experience:

I could sleep for eight hours straight, but I had an incredibly guilty feeling about leaving the mother alone with the child. Additionally, I couldn’t help but feel a little sad, knowing that I might miss out on moments like him burping, yawning or laughing. (Father)

The fathers who were present at the home visit during pregnancy perceived themselves as actively included in the service even before birth: “I’ve been in demand from day one” (Father). However, one father described himself at the clinic as being “a bit passive, carrying the child and equipment, and being a bit of a sidekick” (Father). Fathers also pointed out that inclusion involves more than quickly asking if the father has any questions. A couple of fathers expressed that they would have liked more home visits in addition to the regular consultations at the CHC, and they did not know why they had not received them. Interestingly, the mothers did not outline the same needs.

#### Mastery Process

During the interviews, all the parents expressed their gratitude for becoming a family and that parenting had enriched their lives. The parental role was described as meaningful and as a responsibility: “You can impact and shape another life – a life which in turn is completely dependent on you” (Mother).

Some described the first year as an active and continuous process that followed their child’s development. The first months were the most intensive. Getting to know their child over time, the child’s development around communicating its needs, the introduction and establishment of routines and learning to be more flexible gave parents a feeling of regaining control: “The parental confidence develops over time … you are getting more and more confident and knowing your child better and better” (Mother).

Monitoring the child’s overall well-being, mastering challenging situations, maintaining patience and feeling oneself to be “a safe haven” (Father) for the child served to boost mastery: “I feel like a world champion when I realise what is wrong or manage to turn around a challenging situation” (Mother). Previous and current mastery strategies varied, even within couples. Some parents had high general self-esteem, some were more insecure and others had high expectations around managing things themselves and difficulties with accepting and receiving support. For one mother, it took 6 months before she understood her feelings and sought support. She had not allowed herself to be vulnerable and, retrospectively, would have liked the PHN to have asked her more directly about her needs.

Support from both peers and PHNs was seen as valuable. Meeting other parents was viewed as important, both to create a good, everyday life and to shape their parental role. By learning, sharing and using humour in a safe environment, parents experienced normalcy. Most mothers highly appreciated maternity groups. Indeed, both parents appreciated meeting peers at the CHC and expressed that they would not replace the regular consultations with home visits. One exception was the home visit immediately following the birth, the advantage of which was that the parents did not have to go out with the baby.

The CHS was experienced overall as accessible, and parents appreciated having the opportunity to call, send a text message or drop in at the clinics without fixed appointments. In addition, the clinics were in the parents’ neighbourhood, and time frames for both consultations and home visits were seen as lengthy. Parents expressed that the health checks, weight monitoring and vaccination programme were a form of positive external check, in addition to the parents’ own assessment: “They have been very reassuring and relaxing, they have not been pushy and – how should I put it, (not) hysterical, but helped and allayed our fears a little when we were unsure” (Father).

The PHNs as a group were perceived overall as supportive and patient, despite the fact that some parents had opinions about individual PHN’s suitability. For example, “she was born to be a public health nurse” (Mother) and “maybe she had social inhibitions” (Father). Personal contact, such as when PHNs used their own children as examples or when parents noticed what one mother described as a ‘human touch’, contributed to a good connection. Other experiences that were highlighted included communication based on dialogue, good communication with the child and not appearing judgemental if the child was cranky. In addition, parents emphasised the importance of being accommodated when they asked obvious questions and lacked confidence in their role: “I have often thought ‘What am I doing? I have no idea about this’, but they have been very understanding, and it turned out fine. They seem to be accustomed to dealing with all sorts of parents” (Father).

Two of the families had the same PHN during their pregnancy and throughout the first year postnatally, and those parents highlighted this continuity as unreservedly positive. Others underlined as unfortunate the lack of continuity from having more than one PHN because a common point of reference between the consultations was important – especially when parents were facing challenges.

## Discussion

Overall, the CHS with the integrated NF programme was perceived as providing satisfactory support in the first year postnatally, facilitating the parents’ development of parental confidence. However, we identified some challenges alongside the well-functioning elements which affected the parents’ support needs and their perceived support. We will compare our findings with previous studies and the four sources of SE: enactive mastery experiences, vicarious experiences, verbal persuasion, and physiological and affective states.

First, we will address the need for information about the service and the challenges in defining the parents’ own support needs. We will then discuss the importance of functional co-parenting, the involvement of fathers in the CHS and meeting peers. Finally, we will discuss the duality of receiving support and being checked up on.

### Lack of Public Awareness of the CHS’s Offering: The Home Visit During Pregnancy as a Potential Door Opener

Despite the parents being in a socioeconomically low-risk group and hence our assumption that the parents had a high level of health literacy (Svendsen et al., 2020), we found the parents to have little knowledge of the CHS before they started to use the service. A need for public awareness around pre- and postnatal professional support is found in other studies (Aston et al., 2015; Høgmo et al., 2021; Kaiser et al., 2022) and highlights that new parents would benefit from receiving clear and proactive information about the service (McLeish et al., 2021).

We found that the home visit during pregnancy was appreciated by parents. It facilitated knowledge of the postnatal care pathway, provided information about parenting and initiated the parents’ relationship with the PHN who would provide follow-up postnatally. These elements have a key function in accommodating parents’ needs (Barimani & Vikström, 2015; Haggerty et al., 2003, 2013). However, we identified some organisational challenges, such as having the visit too close to the due date, not receiving information about the visit from GPs or midwives and not reaching out to all fathers. Some parents also described home visits as less clear and more unplanned than consultations at the clinics. This experience may be caused by various factors. Considering that the prenatal home visit is the first meeting, the service is unknown and there is as yet no child, the visits might depend more on the individual PHN’s management and communication skills than the regular consultations at the CHC. These consultations have a clearer agenda, consisting of baby health checks and vaccination. Moreover, McLeish et al. (2021) found that first-time mothers struggled to articulate their problems since everything was unfamiliar and a trusted relationship had not yet been established. These factors also apply to fathers (Høgmo et al., 2021). In addition, some parents might feel vulnerable when exposing a lack of knowledge regarding how to correctly navigate the service, which underlines the need for proactive information provision and management from professionals (McLeish et al., 2021). Cowley et al. (2015) suggest that health visitors need to actively encourage the use of their service, particularly in the first period postnatally when the service is unknown to the recipients.

The CHS postnatal offering consists of several forms of support, including regular consultations, additional consultations at the clinic, a drop-in service, a text message and telephone service and home visits. Our findings indicate the need for clearer and more accurate information about when it is appropriate for parents to ask for postnatal home visits versus other forms of support. The threshold for parents to ask for postnatal home visits seemed high, indicating that home visits were perceived as the most extensive form of support. To lower the threshold for receiving extra support, the NF programme was integrated into the CHS as a universal offering because a universal approach is found to be less stigmatising (City of Oslo, 2018; Leirbakk et al., 2018, 2019; Solberg et al., 2022). However, considering the general lack of awareness around postnatal support, particularly home visits, our findings indicate a need to promote and normalise the utilisation of home visits.

### Defining One’s Own Support Needs May Be Challenging: A Supportive Environment as a Facilitator

McLeish et al. (2020) underlined the importance of receiving accurate information about postnatal services during pregnancy. However, they found first-time mothers’ satisfaction with postnatal care and parental confidence to be more influenced by having their actual postnatal needs for support met, rather than their prenatal expectations of support.

According to our findings, all the parents were overwhelmed and affected by their physiological and affective states, particularly in the first period postnatally. Parenting had made them more sensitive, due to increased feelings of meaningfulness and responsibility. Some parents were reserved around asking for support or struggled with their emotions and needed a more proactive PHN to help them define their needs. However, being proactive as a professional is an exercise in balancing between the parents’ independence and the allocated support. Defining the parents’ needs without their input and giving too much support might actually delay parents’ own involvement and enactive mastery experiences (Sæther et al., 2023) or disaffirm parents’ competence (McLeish et al., 2021). This can be perceived as negative verbal persuasion, thus undermining parental confidence (Cowley et al., 2015). In the interviews, we identified a range of challenges, such as breastfeeding issues, fragmented sleep and strong and fluctuating emotions, which were retrospectively seen as normal by the parents. Addressing these normal challenges might help parents to understand their situation and reassure them around what is normal to ask questions about, as well as enable them to define their needs and eventually take the lead in dialogues with the PHN. Indeed, client-led dialogues are found to facilitate parental confidence (Aston et al., 2015).

Relational continuity plays an important role in building trust and defining needs (Aston et al., 2015; Barimani & Vikström, 2015; Cowley et al., 2015). The parents who had the same PHN throughout the first year appreciated this continuity. Although most participants did not have the same PHN, they nevertheless experienced a kind of relational continuity since their PHNs all shared a common attitude. Overall, the parents perceived the PHNs as being tolerant and friendly, having enough time and being available. Engagement and a non-judgemental attitude from the PHNs, combined with communication based on dialogue, facilitated a supporting and trusting environment. Acknowledgement of parents’ capabilities and equalising the power structure through dialogue may have served as verbal persuasion from PHNs. These characteristics are all found to be important for satisfaction with postnatal support and enhancing parental confidence (Aston et al., 2015; McLeish et al., 2021; Sæther et al., 2023) and to be in line with the intentions of the CHS and NF (City of Oslo, 2018; Norwegian Directorate of Health, 2017).

### Co-Parenting and Paternal Involvement: The Prenatal Visit as a Facilitator

The couples in this study supported and acknowledged each other and offered each other verbal persuasion, all of which are found in functional co-parenting and facilitate PSE (Favez et al., 2016; Feinberg, 2002; Leahy Warren, 2005). In Norway, there are increasing societal expectations concerning fathers’ contribution to childcare, and fathers receive a minimum of 15 weeks of parental leave (Norwegian Labour and Welfare Administration, 2023). Involvement in childcare is found to facilitate parental confidence, which can be related to achieving enactive mastery experiences, as well as establishing a connection with and getting feedback from the child – a form of verbal persuasion (Sæther et al., 2023). In addition, the home visit during pregnancy can be experienced as verbal persuasion if fathers feel supported and involved. A positive appraisal from a significant individual, apart from one’s partner, friends and family, is considered to be of special importance because such appraisal is objective rather than rooted in kindness (McLeish et al., 2021).

Being present at the prenatal visit made fathers feel included and important, a finding supported by Solberg et al. (2022) in their study of 13 fathers receiving an NF home visit during the mothers’ pregnancy. They also found that couples gained a common starting point for further reflection before entering parenthood, and fathers felt a greater sense of equality with mothers when visited at home compared to visiting the clinics. Some fathers in our study expressed a desire for more home visits without specifying a particular reason, except for citing their positive experiences with previous visits. However, their partners did not express the same need, suggesting that fathers’ needs might be overlooked. Fathers’ need for inclusion in the CHS has been addressed in recent studies (Høgmo et al., 2021; Solberg et al., 2022; Solberg & Glavin, 2018; Wells, 2016). These studies highlight that PHNs must have knowledge about the fathers, not just gender-stereotyped opinions (Barimani et al., 2017), and must ensure that support is not given solely to mothers. In Sweden, a series of visits for fathers/non-birthing partners have been implemented in the service to reach out to this group (Odonde et al., 2022).

### Meeting Peers at the CHC Versus Receiving Home Visits

For both mothers and fathers, meeting peers motivated them to visit the CHC. Meeting others in the waiting room at consultations, when using the drop-in service and in the maternity groups, served as important sources of vicarious experiences. The importance of peers in developing confidence in the parental role was prominent among our participants and is supported by previous studies (Barimani et al., 2017; De Sousa Machado et al., 2020; Fletcher & StGeorge, 2011). Most of the study participants did not have their own parents living nearby, which might explain the lack of support from grandparents in the findings. A general lack of vicarious experiences in wider society and poor intergenerational integration in parenting have been identified as challenges regarding PSE (Sæther et al., 2023). This might contribute to the importance of and desire to meet peers during this time. Previous studies have found that facilitating peer meetings is one of the most important interventions the CHS can provide to support mothers in their transition regarding their development of parental confidence (Glavin et al., 2017; Leahy-Warren et al., 2012).

Meeting peers at the CHC and receiving home visits are both found to facilitate parents’ development of parental confidence, but these activities are mutually exclusive. This is not necessarily challenging; rather, it emphasises both the breadth of the support offering and the importance of tailored support and flexibility in providing that support in all its forms. This finding is supported by Cowley et al. (2015), who found that responsive and proportionate services can enable parents to negotiate the different forms of support provision adapted to their needs, which change with time and circumstances.

### Receiving Support and Being Checked Up On

The parents in this study appreciated receiving health checks for the baby and followed the recommended vaccination programme. Reassurance about their child’s health and development is associated with PSE (Sæther et al., 2023). However, the CHS, particularly home visits, was also perceived as an opportunity for the welfare state to check on how children are being cared for. In this study, parents expressed this as appropriate and as an important societal mission. In Solberg et al.’s (2022) study, fathers reflected on whether home visits served a hidden purpose beyond getting to know families and facilitating further follow-up. Apart from being a health-promoting service that supports parents in building their confidence and enhancing their parenting skills, the CHS also has the mandate to prevent, uncover and avert violence, abuse and neglect (Norwegian Directorate of Health, 2017). In the development of the NF programme, the home visit was chosen as the main form of support delivery, with the intention of creating a more equal power structure between the PHN and the family (Leirbakk et al., 2018). The PHNs experienced that building trust was the foundation for all collaboration with the family and found the home visit during pregnancy to positively affect follow-up and continued care (Leirbakk et al., 2023).

Our findings indicate that there might be tension around receiving support whilst knowing that the PHN has a dual mandate. The parents are aware that there is an evaluative component and thus a risk of receiving negative verbal persuasion. Nevertheless, the parents in the present study expressed trust in the CHS and acknowledged the importance of the dual function, finding the offering of home visits positive. However, parental trust in public services might be more disputed in a more diverse sample, as all our participants had a Norwegian or Nordic background. This is exemplified in a recent study which found that immigrant parents felt disempowered in their interactions with the Norwegian welfare system (Tembo et al., 2021).

### Implications for Practice

First-time parents are not a uniform group. However, a common issue for many new parents is that everything is new and unknown. To benefit fully from the CHS, they require clear and proactive information about the service and sometimes even help in defining their needs. The home visit during pregnancy facilitates management and informational and relational continuity (Barimani & Vikström, 2015) and includes the fathers to a greater extent. However, to better address parents’ needs, the service should target paternal involvement, acknowledge the importance of co-parenting and peer support, prioritise relational continuity and provide proactive information about postnatal home visits throughout the first year.

### Strengths and Limitations

We believe that this study’s findings may enhance the knowledge base concerning family-orientated services; in addition, the findings provide insights into fathers’ perspectives, which have been called for (Davison et al., 2017). However, the study sample comprised only 13 participants, all of whom were from a low-risk population. The sample does not represent the diverse population of first-time parents in Oslo. Other limitations include challenges around the retrospective nature of the parents’ experiences and the transferability to countries with different welfare and healthcare systems. Although a retrospective viewpoint poses a risk of inaccurate recollection, it also allows for deeper reflections unaffected by the challenges in the actual period. Additionally, conducting interviews by phone lacked non-verbal communication, but repetition and confirmation of the participants’ statements were carried out during the interviews to ensure credibility. However, after the interviews, we did not involve the participants in further reflections or corrections of their statements. In addition, when coding the data, there is always a risk that context and subjective interactions are not incorporated sufficiently into the analysis (Marvasti, 2019). A final limitation is that all the authors are female, which may be a limitation regarding the perspective of fathers. Three of the four authors, including the interviewer, are PHNs and possess a deep understanding of the CHS, while the remaining author provides an outsider’s perspective. Throughout the analysis and writing phases, we discussed and reflected upon our preconceptions and interpretations of the findings, to provide the clinical field with thoroughly developed knowledge.

## Conclusion

Being a first-time parent is overwhelming, and defining support needs might be challenging, particularly in the first period postnatally. In addition, parents’ support needs, in terms of feeling ‘good enough’ as parents but also in mastering parenting tasks, are constantly changing. This is due to the complexity of interacting factors and the ongoing development of parental confidence. Support from the CHS with the integrated NF programme was experienced overall as accommodating and available, when parents retrospectively reflected on the first year after the birth. The home visit during pregnancy, reassurance and physical check-ups on children’s development and health, the ability to easily make contact, having dialogues with non-judgemental health personnel and meeting peers at the CHC seemed important for the parents’ development of parental confidence. However, we identified a need for more proactive information provision and communication about when and how to use the service, especially regarding postnatal home visits. In addition, attention must be paid to the involvement of fathers and to ensuring continuity of care in the CHS; the importance of functional co-parenting and peer meetings for developing parental confidence must also be acknowledged.
